# Developing public health clinical decision support systems (CDSS) for the outpatient community in New York City: our experience

**DOI:** 10.1186/1471-2458-11-753

**Published:** 2011-09-30

**Authors:** Sam Amirfar, John Taverna, Sheila Anane, Jesse Singer

**Affiliations:** 1 Primary Care Information, Project New York City, Department of Health and Mental Hygiene 42-09 28th St., 12th floor, Long Island City, NY 11101, USA

## Abstract

**Background:**

Developing a clinically relevant set of quality measures that can be effectively used by an electronic health record (EHR) is difficult. Whether it is achieving internal consensus on relevant priority quality measures, communicating to EHR vendors' whose programmers generally lack clinical contextual knowledge, or encouraging implementation of EHR that meaningfully impacts health outcomes, the path is challenging. However, greater transparency of population health, better accountability, and ultimately improved health outcomes is the goal and EHRs afford us a realistic chance of reaching it in a scalable way.

**Method:**

In this article, we summarize our experience as a public health government agency with developing measures for a public health oriented EHR in New York City in partnership with a commercial EHR vendor.

**Results:**

From our experience, there are six key lessons that we share in this article that we believe will dramatically increase the chance of success. First, define the scope and build consensus. Second, get support from executive leadership. Third, find an enthusiastic and competent software partner. Fourth, implement a transparent operational strategy. Fifth, create and test the EHR system with real life scenarios. Last, seek help when you need it.

**Conclusions:**

Despite the challenges, we encourage public health agencies looking to build a similarly focused public health EHR to create one both for improved individual patient as well as the larger population health.

## Background

In 2004, in an effort to help New Yorkers live longer and healthier lives, the New York City Department of Health and Mental Hygiene (DOHMH) undertook a policy initiative called Take Care New York (TCNY). The goal of TCNY is to improve population health by helping New Yorkers take discrete and identifiable steps to improve their health through focusing on ten items ranging from prevention to health screening and chronic disease management. After identifying these ten areas (see Table 1), the DOHMH, guided by then Commissioner Thomas Frieden, tracked these TCNY measures to monitor the city's progress [1]. In early 2005, the Commissioner also set up a Taskforce at the DOHMH, the Primary Care Information Project (PCIP), tasked with reducing health care disparities among New Yorkers, especially those living in lower socioeconomic neighborhoods, through health information technology. PCIP was established to help low-income, disadvantaged communities and the providers who deliver health care to these communities take advantage of technological innovations in order to improve the quality of care of their patients as measured by the ten TCNY measures [2]. Recognizing the opportunity to improve care and collect data on the TCNY defined clinical areas, PCIP, in collaboration with our selected EHR vendor, developed the corresponding TCNY quality measures for use at the point of care. These measures at the point of care became the basis of our clinical decision support systems (CDSS). Within the EHR, these CDSS alerts inform the provider by a series of prompts on their computer screen that the patient they are currently seeing in their office is due for specific health maintenance screenings or chronic disease management based on the patient's age, gender, and comorbidities.

**Table 1:** Summary Table of Take Care New York Indicators

TCNY Agenda Item	Indicator
**1. Have a Regular Doctor or Other Health Care Provider**	Adult New Yorkers without a regular doctor
**2. Be Tobacco Free**	Adult New Yorkers who smoke
**3. Have a Healthy Heart**	Proportion of New Yorkers with high blood pressure and cholesterol. Proportion of New Yorkers with well-controlled hypertension, cholesterol & diabetes
**4. Know Your HIV Status**	Number of New Yorkers who die from HIV/AIDS
**5. Get Help for Depression**	Prevalence of untreated depression
**6. Live Free of Alcohol or Drugs**	Alcohol-attributable mortality Drug-related deaths
**7. Get Checked for Cancer**	Screening rates for breast cancer Screening rates for cervical cancer Screening rates for colon cancer
**8. Get the Immunizations You Need**	Influenza immunizations among New Yorkers age 65+
**9. Make Your Home Safe & Healthy**	Women who die from intimate partner homicide Children with newly-identified blood lead levels (BLL) > 15 μg/dl and an identified lead-based paint hazard
**10. Have a Healthy Baby**	Infant mortality rate per 1, 000 live births

This collaborative development between a government entity and a private EHR vendor resulted in a unique public health oriented electronic health record, which has actionable quality measures that prompt providers to take steps for preventive care or chronic disease management for various health issues targeted within New York City. A public health oriented EHR is an electronic health record system which in addition to enabling the provider to provide appropriate healthcare resources to the care of their patients, allows for safe, convenient, and timely transfer of public health information bilaterally between providers and their local and state public health organizations and departments. To contribute to the measurement of the TCNY measures, the aggregate values of the corresponding measures are transmitted monthly to DOHMH. Analysis of such data allows for improved efficacy of important public health programs such as infectious disease surveillance, chronic disease prevalence studies, and environmental exposures, to name a few. This method of gathering data from community providers has the potential of eventually replacing the traditional slow and incomplete methods of data collection, such as chart reviews, which public health agencies have been using for more than 100 years. Since we developed this public health oriented EHR, we have implemented the system to approximately 2600 providers in the five boroughs of New York City representing approximately 2.5 million patients. In 2010 alone, our CDSS alerts have been involved in million patient encounters. While the EHR development process has been long, the details of the journey may help illuminate the pathway for other local or state governments with a similar interest.

## Methods

PCIP considers electronic health records to be one of the best and most scalable methods of helping community providers improve the care they provide while containing providers' operational costs in the long term after an initial upfront investment [3,4]. EHRs allow documentation of a clinical encounter in a structured codified format with electronic ordering and recording of laboratory tests, radiologic examinations, immunizations, vital signs, medication and allergies to name but a few important functionalities necessary for a clinical encounter. Structured data is the crux of public health functionality because it enables: quality measures to assess ambulatory care quality, CDSS to communicate best clinical practices to providers, and public health interfaces to link with existing DOHMH systems. All three of these functionalities are at the core of an ideal public health-oriented EHR, and all three are enabled by structured data. At the time of PCIP's establishment, there was no EHR that included the public health functionality that we envisioned.

Before implementing EHR systems in the community, the DOHMH tried to take advantage of existing technological innovations to improve the disparity of health care through the distribution of personal digital assistants (PDA) to physicians working in federally-funded community health centers [5]. The PDAs were primarily used for electronic prescribing, while health alerts and information on emergency preparedness were available for download via a website accessible from the devices and maintained by an outside vendor. These health alerts were an early, primitive form of CDSS. They informed providers of potentially relevant information in hopes of helping them make an informed clinical decision. However, even though vendor-led training was conducted during the implementation phase, it quickly became apparent that adoption even of a seemingly easier technological implementation than a full electronic health record can be difficult, particularly when the methodology used is outside of the standard workflow of a physician [6-8]. When the complexity of implementing PDA-based CDSS to providers approached the anticipated difficulty of implementing EHR-based CDSS, we decided to concentrate our efforts on a more comprehensive EHR in order to gain the added benefits over a simple electronic prescribing software. This initial project provided us the insights to many of the barriers and complexities of technological implementations in clinical settings such as provider comfort with new and existing technology, software and hardware support and maintenance required, length and importance of user training, and provider and staff "buy in" in the process. As a result, we sought to identify an EHR vendor that would collaboratively develop our set of public health requirements.

In January of 2006, the DOHMH released a Request for Proposals (RFP) to identify a qualified vendor to provide an electronic health record system to our New York City community-based providers as well as to Correctional Health Services, the City entity responsible for providing health care in the New York City's jail system. After the release of the RFP, six vendors were invited to provide a live demonstration of their product to the DOHMH product review committee, made up of the Assistant Commissioner of PCIP, Chief of Operations of PCIP, Director of Finance and Operations of Health Care Access and Improvement, and Director of Vendors and Technology Partners. The criteria used in the vendor selection process included the size of the company, years the company has been in business, stability of the company determined by a stable leadership team over time, the usability of the product as determined through impromptu case presentations to the vendor, ease of use, adaptability, and the interest and resources available to the company to make the necessary changes that we were seeking. The selection of eClinicalWorks as the awardee of the four-year contract was announced in the fall of 2006. eClinicalWorks was chosen because of their commitment to being our partner in building an affordable public health-oriented EHR. After selection, we soon began development discussions in which we communicated necessary public health requirements to the eClinicalWorks developers.

Once the EHR vendor had been identified, PCIP defined the necessary public health requirements and categorized them into two development cycles. The first development cycle would produce the CDSS alerts as well as the corresponding order sets which would facilitate providers ordering. In addition, during the first development cycle, a more robust registry system that would allow providers to assess their patient population on demand would be developed. The first development cycle involved three phases, which are pre-development discussions, collaborative development, and quality assurance [see Table 2]. The second cycle focused on developing functionality that will allow the DOHMH to distribute public health alerts during important events, such as infectious disease outbreaks.

**Table 2:** Phases of Development Cycle

Step	Goal
Pre-development	• Involve stakeholders
	• Build consensus

Collaborative Development	• Plan frequent meetings
	• Develop clinical to programmatic translation

Quality Assurance	• Ensure alpha testing
	• Provide beta and clinical content testing

In parallel to working on the Request for Proposals, PCIP started work on translating the ten TCNY priority measures into actionable CDSS alerts at the point of care. These CDSS alerts have been defined as "an automated process for comparing patient-specific characteristics against a computerized knowledge base with resulting recommendations or reminders presented to the provider at the time of clinical decision making" [9] That is, making known to the physician a patient's quality measure status at the point of care is the decision support. We expanded this standard function of CDSS by making it actionable for our providers so that not only are providers aware of the applicable alert, they can also address the clinical need with one click of a computer mouse. However, the first step in producing these new alerts was to investigate the existing measures at that time and see how they were consistent with the standards produced by other national and state organizations.

Our approach to developing electronic CDSS measures involved consulting existing national bodies as well as internal DOHMH subject matter experts in order to translate their retrospective paper measures to point-of-care electronic measures. We examined existing measures produced by national organizations such as the Physician Quality Reporting Initiative and National Committee for Quality Assurance as well as measures produced by independent national and professional medical associations such as the U.S. Preventive Services Task Force, American College of Cardiology, American Diabetes Association, and American Lung Association.

In addition to reconciling with national standards, we invited DOHMH subject matter experts on sexually transmitted diseases, tobacco, asthma, and mental health to get their input on the respective measures and to devise the best method of data collection. We met with health organizations within New York City to get their recommendations on a practical set of measure alerts. For example, PCIP worked with subject matter experts, informatics specialists, and clinical providers from the Institute for Family Health, a community health center based in New York City, to develop our measures. These measures needed to incorporate clinical guidelines, population level epidemiologic information, and optimal design based on extensive clinical experience to ensure that they were not only clinically appropriate and useful to clinicians but were also consonant with existing workflows to ensure their use. At the end of this one year process and after incorporating the recommendations of all these subject matter experts, we had transformed the 10 TCNY general health areas into 40 specific quality measures at the point-of-care CDSS alerts (see Table 3).

**Table 3:** All Alerts are on an annual basis unless specified otherwise

TCNY Domain	Measure Name	Brief Description of Measure
Have a regular doctor	Patients see assigned PCG	See assigned PCG at least once
Be tobacco free	Smoking status	Update smoking status
	Smoking cessation intervention	Stop smokers with medications or counseling
Keep your heart healthy	BP control in HTN	Control BP < 140/90 in patients with HTN without DM or IVD
	Antithrombic	Provide Patients with IVD or DM with antithrombic therapy
	Body Mass Index	Measure the height and weight for BMI
	Cholesterol screen	Screen patients without DM or IVD for cholesterol/HDL
	Cholesterol control	Control patients without DM or IVD to a total cholesterol < 240 or LDL < 160
	LDL control (high risk)	Control patients without DM or IVD to an LDL < 100
	LDL testing (high risk)	LDL screen for patients with DM or IVD
	A1C testing	Measure at least one HbA1c in DM patients every 6 months
	A1C control (< 7%)	Control HbA1c below 7.0% in DM patients
	BP control in IVD	Control BP < 140/90 with a diagnosis of IVD without DM
	BP control in IVD AND HTN	Control BP < 140/90 with a diagnosis of IVD AND HTN without DM
	BP control in DM	Control BP < 130/80 with a diagnosis of DM
	BP control in DM AND HTN	Control BP < 130/80 with a diagnosis of DM AND HTN
Know your HIV status	HIV screening	Screen for HIV
	HIV viral load and CD4	Monitor HIV patients' viral load and CD4 every 3 months
Get help for depression	Depression screening	Screen patients for depression
	Depression follow-up	Monitor patients with depression every 3 months
	Depression control	Lower depressed patients PHQ-9 score
Live free of dependence on alcohol and drugs	Alcohol use screening	Screen for alcohol misuse
Get checked for cancer	Colorectal cancer screening	Screen 50-80 years old patients
	Breast cancer screening	Screen female ≥ 40 years old
	Cervical cancer screening	Screen female 18-64 years old
Get the immunizations you need	Influenza vaccine (child)	Vaccinate children 7 months to 5 years old
	Influenza vaccine (high risk)	Vaccinate high risk patients 5-49 years old
	Influenza vaccine	Vaccinate > 50 years old
	Pneumococcal vaccine	Vaccinate ≥ 65 years old or ( ≥ 60 months AND in a high risk group)
Make your home safe and healthy	Lead testing (1 year)	Screen one-year old with a blood lead test
	Lead testing (2 years)	Screen two-year old with a blood lead test
	Asthma symptom assessment	Assess 18-56 years old for asthma severity
	Asthma control	Assess 5-56 years old with persistent asthma who were prescribed appropriate medications
Have a healthy baby	Chlamydia screening	Screen eligible female patients
	Sexual history	Update > 18 years old sexual history
	Sexual history taken	Update 12-17 years old sexual history

Once these 40 clinical quality measures were agreed upon, the PCIP development team undertook the task of translating these 40 measures into logic models that could be processed and interpreted by a computer program. The logic models were specific actions and processes we required the computer program to run for every patient depending on a variety of demographic and clinical content entered by the provider in the EHR. For example, if the patient was over 18 years old, we required the program to identify if smoking status was documented for the patient and if it had, to determine if the last status of "never", "former", or "current smoker" had been updated within the past year. The creation of the logic models was a good method that allowed the team to identify how the EHR system could determine the particular measures a patient qualified for. It also helped formulate in our minds the necessary data elements that were crucial for a particular measure. In addition, the process of creating the logic models was successful in building consensus across subject experts on how a measure can be interpreted proactively at the point of care and how to reconcile best practices with small practice workflow.

However, translating guidelines at the point of care often resulted in these logic models becoming extremely complicated. For example, the EHR software would have to keep track of each patient through the logic diagram, save their progress, and retrieve it quickly at the next clinical encounter. Moreover, the system was then required to keep track of several conditions and to use data for multiple "if-then" statements. It quickly became apparent that this longitudinal examination of the patient was too complicated and these difficulties with complex coding would result in slower system performance. A limitation of the logic models was that its use did not allow for comparative analysis across patients and providers. With logic models, it was simply a matter of where a patient was placed in the flow diagram; there was no inherent "good" or "bad" state for the patient to be evaluated on. While logic models are very good for providing decision support for *one *patient at the point of care, there was no easy way to determine a *group *of patients' compliance to a particular measure. In other words, while the alerts were appropriate and timely, it was difficult to evaluate the compliance rate to these alerts by patient and provider. Despite the complexity and ultimate abandoning of the logic model concept, the method helped to elucidate the data elements necessary to calculate quality measures solely based on EHR derived data.

In response to the challenges presented by the logic model, the team shifted our philosophy to aggregate counting and developed compliant binary counts. Compliant binary counts are defined as true/false assessments of whether a patient meets or does not meet the defined criteria, thus indicating that a patient is either compliant or non-compliant for a particular measure. If a patient meets the criteria for disease evaluation, then a "1" is placed in the denominator. If a patient meets the criteria for measure satisfaction, then a "1" is placed in the numerator. Therefore, every patient for each applicable measure is either, "0/1" or 0% for non-compliant or "1/1" or 100% for compliant. For example, one of our measures asks that every patient with diabetes mellitus have their hemoglobin A1c checked at least once every 6 months and the result to be entered in the EHR. If a patient is documented in the EHR as having diabetes mellitus as a medical condition in their problem list, then they are added to the hemoglobin A1c testing measure and the denominator of that measure increases by 1. If that provider then orders a hemoglobin A1c test for the patient and the result of the test enters into the EHR, the numerator increases by 1 and the patient is fully compliant (1/1) for this measure. If not, the patient is not compliant (0/1). If the provider has 500 diabetic patients, this number can be summed for a compliance rate for this measure. If 400 hemoglobin A1c lab tests results are eventually entered for these patients, then the provider's compliance rate will be 80%, indicating that 80% (400/500) of the provider's diabetic patients have had the appropriate intervention for this measure. In this way, results from a group of patients can be aggregated by provider, thus providing a quality assessment of the provider's patient panel which for this particular provider for this particular CDSS alert would be a compliance rate of 80%. The major advantage of using binary counts is that it allows a quantitative assessment of each patients' compliance to a particular CDSS alert and allows for comparison of providers' performance to city or zip code averages.

In order to provide some lag time between the time the provider orders a lab and the result return from the commercial laboratory, we also introduced the concept of snoozing alerts. It is a transition phase allowing the patients a certain amount of time (usually one month) to get their labs drawn and for the result to return to the provider's EHR. A snoozed alert disappears from a provider's CDSS menu but will return as an alert if the lab result does not appear in the time window.

Once measures had been defined and we had agreed on how the DOHMH and our providers would use them to monitor and improve important quality indicators, PCIP began the development phase in which we created an overall plan for customizing the electronic health record to meet the needs of New York City providers. We developed a timeline for the project and defined our measures for success, namely 2000 providers being live with a full public health oriented EHR with actionable CDSS in two years. Our long term goal has always been to improve clinical outcomes for New Yorkers and all our project plans were produced with this overarching goal in mind.

The next crucial step in this phase was producing our CDSS alerts within the software by translating the measures with the binary counts we had produced with our DOHMH and community partners into definitions that computer software could understand. To do this, we had to create a PCIP team dedicated to producing this new system. Our development team consists of quality assurance specialists who test the new product, EHR super users who go to the providers and provide tailored training, physicians who provide clinical input and practice workflow experience, epidemiologists who analyze the resulting data, and project managers who coordinate all the activities. Once the team had been established, the operational structure for collaborative development between PCIP and eCW was created. We agreed that meetings between ourselves and eCW software developers had to be frequent and lengthy in order to solidify relationships and to make sure we had similar expectations of the final product. During these meetings, we spent the majority of the time defining the scope for each new proposed functionality as well as the rationale behind its introduction. We felt it was important that the vendor had "buy-in" for each functionality and felt that the proposed changes were necessary as well. Having senior executive leaders with both vision and content matter expertise closely involved was critical because it created an environment where both the DOHMH and vendor could overcome the inevitable obstacles. The cooperation and shared vision helped both organizations to succeed and achieve their respective goals.

Even with the time spent describing each new functionality during the collaborative development meetings, there were many follow-up discussions for clarifying or redeveloping functionalities because items that eCW developed were not always what we had in mind. We quickly came to realize another important aspect of our relationship. While we might be agreeing on the *facts*, the *interpretation *can also make or break a function in terms of usefulness, effectiveness, and adoption. The software developer approaches each solution with a practical engineering viewpoint that must be countered with a clinical or public health viewpoint for balance. Because of this, it is very important to get frequent visual updates for each functionality via demonstrations to make sure the vision does not veer off the path from what is expected.

One of the issues that our collaborative development team needed to discuss was the use of technological standards. Standards are necessary in an environment where the software needs to exchange medical content entered in the progress notes across many users. The software vendor needs standards so that their system is able to communicate between different users. For the DOHMH, our scope is broader since we need standards to satisfy our larger health information technology needs in order to receive aggregate data transmissions of clinical information both among all electronic health users in New York City and to the DOHMH. Since the decision support tools were implemented for all our providers, it was necessary to discuss the standard set of data the software would utilize to produce the alerts with the EHR vendor. Some standards already existed and we simply used them in our CDSS programming. For example, we used the International Classification of Diseases (*ICD-9) *codes as the standard for codifying the patient's medical problems and Logical Observation Identifiers Names and Codes (LOINC) codes for lab results returning from commercial laboratories. For other data elements where no standard exists, it was necessary to make our own. Laboratory ordering was one area where there are no established standards and we made our own numerical classification system called community IDs. For example, an HIV test ordered from the system is the same irrespective of the commercial laboratory where the test is ordered.

## Results

Once the different CDSS functionalities had been developed in the electronic health record, testing the functionality began in earnest. We had underestimated the time and effort that testing of the functionalities would require. In fact, testing of the measures required more resources and was more complex than the development phase. Between physically entering test patients, ensuring that CDSS measures were functional, and keeping track of the bugs and versions developed, we quickly realized we had underestimated this portion of CDSS development.

Testing of the various bugs within the system compromised two main stages. The first stage involved making sure that the functionalities of the CDSS system were working. This included alpha testing which entailed making sure new CDSS functions in the system worked such as quick links to labs and medications, and that there were no gross and obvious system errors. One of the most common errors we discovered during this part of the testing were usually HTML or JavaScript errors where the CDSS alerts were supposed to be. As these areas in the system were slowly corrected, we focused our attention on the second stage of quality assurance. In the second stage, we primarily focused upon correcting any clinical content errors such as clinical alerts that appeared for the wrong patients using use cases. For example, if a test hypertension patient in the system had a blood pressure of 135/85, a blood pressure alert would not appear for this particular patient. However if a diagnosis of diabetes was entered for this patient, their blood pressure would qualify them for the corresponding more stringent blood pressure alert for a diabetic patient.

## Conclusions

Building meaningful measures as CDSS and incorporating them effectively into an electronic health record was more challenging than anticipated. The steps involved are difficult and long and really cannot be shortchanged. All view points, including those of public health, clinical, and technical must be represented so that there is consensus. Since these measures will affect and direct provider workflow, the chosen measures must be clinically significant. There needs to be some understanding of where these measures fit into the other state and national measures that have already been developed. Once the measures are finalized, the difficult part is to translate them into a format that a computer program can understand and utilize effectively. Public health and quality measurement experts must find their counterparts in software engineers who share a public health vision and will prioritize the development and implementation of these measures. During their development, there need to be countless conversations to facilitate transparent translation of the measures from the clinical perspective to an engineering/programming perspective. Finally, testing of the measures needs to be done to make sure that they work at the right time for the right patient to give the right message.

Having completed this process, there are some recommendations we can make to improve the chances of success for others seeking to build CDSS within their EHR. First, organize a workgroup of various clinical experts to help define the scope and build consensus. Sometimes, you will not know how big a project you are facing until you can see the challenge from every perspective. A large pool of experts will help with this. Not only will a large pool of experts allow for a wider viewpoint on clinical measures complying with both domestic and international standards but will also help the project to become a success since more people will have a vested interest in the project. Second, get support for your activities from executive leadership. The full support of two sequential City Health Commissioners and the Chief Executive Officer of our partner software company has been invaluable for getting help and cooperation from not only our respective organizations but from the community as well. Third, find an enthusiastic and energetic software partner who shares your vision for your project and is well prepared to undertake a complex task such as this. The energy in a good relationship can be used to pull both of you through during the inevitable tough times. Fourth, implement a transparent operational strategy so both sides can understand what the other is developing. The same scope and function may be agreed upon in meetings but its actual implementation will inevitably vary. An engineer will always find ways of interpreting facts differently from what a clinician envisions. Do not assume anything except that they will see things differently. It would be unfortunate to find out several months into development that the vendor is producing a functionality that one cannot use. Fifth, create and share use cases. You need to make sure that the right alerts show up for the right patients at the appropriate time. Testing by users and developers guarantees that the new functionality is working properly, but only a clinician can ultimately decide if the software "makes sense" in a clinical setting. Lastly, respect your time and effort in doing this project and ask for help if you need it. Others have gone through the process, so there is no need to reinvent the wheel. Guidance and advice from others can go a long way to getting things back on track. By following these recommendations, one can improve their chances for developing a clinically useful CDSS alerting system while minimizing the inevitable obstacles and frustrations. We have followed these recommendations ourselves in a later phase while developing new functionalities. The process has been much improved: shorter, more focused, and more satisfying for both parties. These efforts of developing a public health oriented CDSS has allowed us to communicate more effectively with providers when they are seeing patients and has advanced the relationship between local government agency and the providers of healthcare into the 21^st ^century.

## Abbreviations

BP: blood pressure; PCG: primary care giver; DM: diabetes mellitus; HTN: hypertension; IVD: ischemic vascular disease; HbA1c: hemoglobin A1c.

## Competing interests

The authors declare that they have no competing interests.

## Authors' contributions

SA literature review and drafted the majority of the manuscript. SA drafted parts of the manuscript and performed major editing. JT performed literature review and wrote parts of the manuscript. JS drafted parts of the manuscript and performed editing. All authors were involved in the conceptual framework and design of the article. All authors read and approved the final manuscript.

## Author's information

All authors work at the Primary Care Information Project (PCIP) which is a bureau of the New York City Department of Health and Mental Hygiene. PCIP is tasked with addressing healthcare disparities in New York City by helping providers, especially those working in lower socioeconomic neighborhoods, with adopting information technology. Since 2008, PCIP and later serving as a Regional Extension Center partner, NYC REACH, has helped over 2500 providers to adopt electronic health records. Jesse Singer is the Executive Director of the Development Group in charge of customizing the EHRs to be more public health focused. Sheila Anane serves as the project manager. John Taverna is in charge of the information technology issues. Sam Amirfar is in charge of updating and modifying the medical content within the EHRs.

## Pre-publication history

The pre-publication history for this paper can be accessed here:

http://www.biomedcentral.com/1471-2458/11/753/prepub
